# Interplay Between Intracellular Ca^2+^ Oscillations and Ca^2+^-stimulated Mitochondrial Metabolism

**DOI:** 10.1038/srep19316

**Published:** 2016-01-18

**Authors:** Benjamin Wacquier, Laurent Combettes, Guy Tran Van Nhieu, Geneviève Dupont

**Affiliations:** 1 Unité de Chronobiologie Théorique, Université Libre de Bruxelles, CP231, Boulevard du Triomphe, 1050, Brussels, Belgium; 2 Université Paris Sud, UMRS1174, Orsay F-91405, France; 3 Institut National de la Santé et de la Recherche Médicale (Inserm), UMRS1174, Orsay F-91405, France; 4 Equipe Communication Intercellulaire et Infections Microbiennes, Centre de Recherche Interdisciplinaire en Biologie(CIRB), Collège de France, 11 Place Marcelin Berthelot, Paris 75005, France; 5 Inserm, U1050, Paris 75005, France; 6 Centre national de la Recherche Scientifique (CNRS), UMR7241, Paris 75005, France; 7 MEMOLIFE Laboratory of excellence and Paris Sciences et Lettres, Paris 75005, France

## Abstract

Oscillations of cytosolic Ca^2+^ concentration are a widespread mode of signalling. Oscillatory spikes rely on repetitive exchanges of Ca^2+^ between the endoplasmic reticulum (ER) and the cytosol, due to the regulation of inositol 1,4,5-trisphosphate receptors. Mitochondria also sequester and release Ca^2+^, thus affecting Ca^2+^ signalling. Mitochondrial Ca^2+^ activates key enzymes involved in ATP synthesis. We propose a new integrative model for Ca^2+^ signalling and mitochondrial metabolism in electrically non-excitable cells. The model accounts for (1) the phase relationship of the Ca^2+^ changes in the cytosol, the ER and mitochondria, (2) the dynamics of mitochondrial metabolites in response to cytosolic Ca^2+^ changes, and (3) the impacts of cytosol/mitochondria Ca^2+^ exchanges and of mitochondrial metabolism on Ca^2+^ oscillations. Simulations predict that as expected, oscillations are slowed down by decreasing the rate of Ca^2+^ efflux from mitochondria, but also by decreasing the rate of Ca^2+^ influx through the mitochondrial Ca^2+^ uniporter (MCU). These predictions were experimentally validated by inhibiting MCU expression. Despite the highly non-linear character of Ca^2+^ dynamics and mitochondrial metabolism, bioenergetics were found to be robust with respect to changes in frequency and amplitude of Ca^2+^ oscillations.

In most organisms, mitochondria play an important role in ATP production and act as Ca^2+^ stores, both functions of these organelles being tightly connected. Mitochondria sequester and release Ca^2+^, thereby affecting the shape, the frequency and the amplitude of the Ca^2+^ spikes in the cytosol^1^,^2^,^3^. In turn, increased mitochondrial Ca^2+^ ([Ca^2+^]_m_) linked to the transfer of Ca^2+^ from the cytosol to mitochondria during [Ca^2+^]_c_ signals stimulates mitochondrial metabolism and allows the coupling of ATP supply with energy demand^4^,^5^,^6^,^7^.

At rest, [Ca^2+^]_m_ and [Ca^2+^]_c_ are similar, in the 100 nM range^8^. Upon cell stimulation by an agonist, inositol 1,4,5-trisphosphate (IP_3_) is produced and triggers cytosolic Ca^2+^ oscillations^9^. In non-excitable cells, these oscillations are due to a cyclical exchange of Ca^2+^ between the cytosol and the endoplasmic reticulum (ER), through the biphasic regulation of the IP_3_ receptor (IP_3_R) by cytosolic Ca^2+^. Fast activation and slow inhibition of the opening of the IP_3_R by Ca^2+^ indeed suffice to generate either Ca^2+^ oscillations in classical deterministic models or repetitive spiking, if noise is considered to play a predominant role in cellular Ca^2+^ dynamics^10^,^11^,^12^. Ca^2+^ entry into mitochondria occurs through a multistep mechanism. By extruding protons out of mitochondria, the respiratory chain creates a large inside-negative potential difference across the inner mitochondrial membrane. This ΔΨ, which is harnessed by the ATP synthase for the production of ATP, allows the Mitochondrial Calcium Uniporter (MCU) to transport Ca^2+^ inside mitochondria^13^,^14^. Ca^2+^ entry then depolarizes the mitochondria, thus reducing its own driving force. When [Ca^2+^]_c_ returns to its basal value, extrusion of Ca^2+^ out of mitochondria occurs through both a Na^+^-Ca^2+^ exchanger (NCX) and a H^+^-Ca^2+^ exchanger, possibly identified as LETM1, although the contribution of this channel to mitochondrial Ca^2+^ transport is not yet firmly established^15^,^16^,^17^.

Uptake by mitochondria of pyruvate, the end product of cytosolic glycolysis, is at the onset of the oxidative phosphorylation cascade. A pyruvate dehydrogenase transforms substrates into acetyl-CoA which enters the Krebs cycle, also called the acid citric cycle or tricarboxylic acid cycle (TCA). This 9-step cycle converts the chemical energy of pyruvate into the reducing power of NADH. In addition, the activity of the malate-aspartate shuttle (MAS) also increases mitochondrial NADH^18^. NADH then feeds the oxidative phosphorylation pathway in which electrons are transferred and finally used to extrude protons and establish a proton gradient between the intermembrane space and the interior of mitochondria. This electrochemical source of energy is then harnessed by the F_1_F_o_-ATPase to phosphorylate ADP into ATP. An increase in [Ca^2+^]_m_ activates metabolism as pyruvate dehydrogenase and two rate-limiting enzymes of the TCA cycle, isocitrate dehydrogenase and *α*-ketoglutarate dehydrogenase, are up-regulated by Ca^2+^ 19. Thus, upon stimulation, the transfer of Ca^2+^ from the cytosol into mitochondria allows for the enhancement of mitochondrial ATP production by the F_1_F_o_-ATPase. Cytosolic Ca^2+^ also directly influences mitochondrial metabolism as a component of the MAS, the aspartate-glutamate carrier (AGC), is stimulated by modest increases in cytosolic Ca^2+^ 18. Given the complexity and highly non-linear character of Ca^2+^ and mitochondrial dynamics, it is useful to resort to computational modelling to clarify their interplay. Several models have been proposed. Many of them focus on mitochondrial metabolism, but less on the effect of this metabolism on Ca^2+^ signalling^20^,^21^,^22^,^23^,^24^,^25^. Other models in contrast, extend computational descriptions of ER-cytosolic Ca^2+^ exchanges to incorporate Ca^2+^ handling by mitochondria. These models shed new light on important specific questions, such as the effect of mitochondria on the amplitude of the Ca^2+^ spikes^26^, the effect of glucose on the frequency of Ca^2+^ oscillations^27^, the modulation of NADH metabolism in pancreatic *β*-cells^28^, the mechanism responsible for Ca^2+^ wave propagation in mitochondrial suspensions^29^, or the importance of the distance between the ER membranes and mitochondria for their cross talk^30^.

Here we extend these previous studies to propose a model accounting for a variety of already published experimental observations centred on Ca^2+^ in non-excitable cells. These observations concern the effect of Ca^2+^ on mitochondrial metabolism, as well as changes in cytosolic Ca^2+^ dynamics occurring when the kinetic properties of the mitochondria are modified. The model is mainly built from the combination of previously published kinetic expressions. One of our aims is to clarify this complex issue by using simple expressions when possible and by motivating the resort to complex mathematical expressions when necessary. This is for example the case for the kinetics of the MCU that we analysed in details. We also found that a reversible Ca^2+^ leak between the cytosol and the mitochondria, which could correspond to the low conductance mode of the mitochondrial permeability transition pore, was necessary to account for a number of experimental observations. The phase relationship between Ca^2+^ peaks in the cytosol, the ER, and the mitochondria^31^ was investigated in details. The effect of modifying the activity of the MCU or of the NCX was next analysed in the simulations, with respect to previous experimental observations in HeLa cells^32^,^33^.

We found that increasing the activity of the MCU first increases and then decreases the frequency of Ca^2+^ oscillations. We tested this counter-intuitive prediction by silencing the MCU in HeLa cells. We then switched to the validation of the model concerning mitochondrial metabolism by comparing computationally obtained time series with the evolution of NADH^4^, voltage difference across the inner mitochondrial membrane (ΔΨ)^34^, and ATP in response to a single Ca^2+^ spike^34^ or in response to sustained Ca^2+^ oscillations^4^,^35^. This validated model could then be used to investigate the sensitivity of mitochondrial metabolism to changes in the frequency and amplitude of cytosolic Ca^2+^ spikes. Besides a strong robustness of mitochondrial metabolism with respect to the characteristics of the cytosolic Ca^2+^ changes, the model predicted that ATP synthesis by mitochondria is most efficient for frequencies and amplitudes of Ca^2+^ spikes usually observed experimentally.

## Methods

### Model

**Figure 1:**
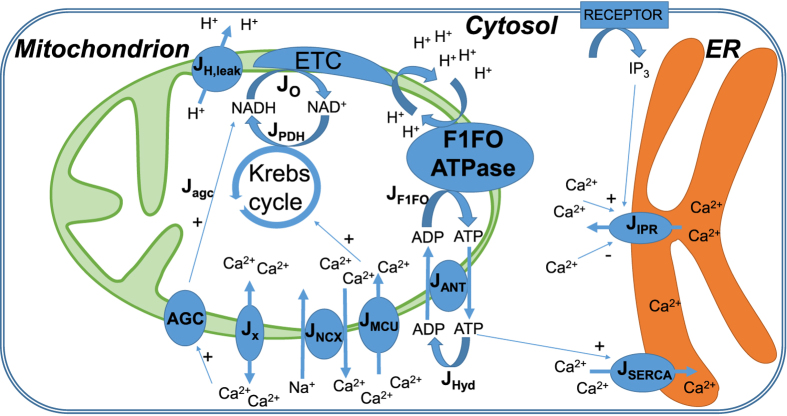
Schematic representation of the model for Ca^2+^ dynamics and mitochondrial metabolism. Non standard abbreviations: J_o_: rate of NADH oxidation; J_PDH_: rate of NADH production by the pyruvate dehydrogenase, followed by the Krebs cycle; J_ANT_: rate of the ATP/ADP translocator; J_x_: bidirectional Ca^2+^ leak between the cytosol and mitochondria (model hypothesis); J_IPR_: Ca^2+^ flux through the IP_3_ receptor. J_AGC_: rate of NADH production induced by the MAS NADH shuttle. See text.

The processes considered in the model are schematized in Fig. 1. Fluxes and reactions are described by ordinary differential equations. As we neglect spatial aspects, we do not consider Ca^2+^ microdomains. More specifically, we do not explicitly incorporate MAM (mitochondria-associated ER membranes) in the model^36^ and focus on cellular average behaviour. All concentrations (including Michaelis-Menten constants) are thus averages on the volume of a given intracellular compartment: cytosol (c), endoplasmic reticulum (ER) or mitochondria (m). Thus, the fluxes are scaled by the appropriate volume ratio when necessary (see the legend of Table 1 for a more accurate description).

We adopt a fully deterministic approach, which is appropriate to obtain predictions about the average effect of the different individual fluxes on Ca^2+^ dynamics^37^ and mitochondrial metabolism. As described in previous approaches^27^,^28^, there is no distinction between the inter-membrane mitochondrial space and the cytosol, as the outer membrane is highly permeable to Ca^2+^ and H^+^. The model is defined by 7 evolution equations and 4 conservation relations given here below (Eqs. (1, 2, 3, 4, 5, 6, 7, 8, 9, 10, 11)). The fluxes appearing in these equations are defined by Eqs. (12, 13, 14, 15, 16, 17, 18, 19, 20, 21, 22, 23).

#### Evolution equations:

- Cytosolic Ca^2+^ concentration
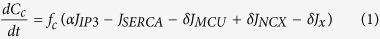
- Using the framework of rapid buffering approximation^38^, f_c_ is the Ca^2+^ buffering capacity of the cytosol. The different volumes of the compartments are taken into account via *α* = V_ER_/V_c_ and *δ* = V_m_/V_c_. J_x_ is a bidirectional Ca^2+^ leak (see the ‘Results’ section for the rationale of this term). Ca^2+^ exchanges with the extracellular medium are not taken into account since they do not play a major role in the interplay between cytosolic and mitochondrial Ca^2+^ during the early spikes following stimulation^31^. Also, we do not consider Ca^2+^ fluxes through the mitochondrial Ca^2+^/H^+^ exchanger. Although this is not firmly established^17^, the Ca^2+^/H^+^ exchanger is thought to correspond to the LETM1 protein^39^ and the levels of expression of LETM1 appear to be inconsequential for mitochondrial Ca^2+^ export during physiological stimulation of HeLa cells^17^.
- Mitochondrial Ca^2+^ concentration
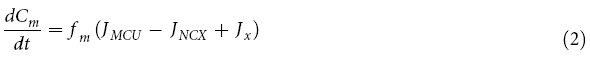
f_m_ is the Ca^2+^ buffering capacity of mitochondria.
- Fraction of inactivated IP_3_ receptors
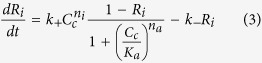
 This equation reflects the biphasic regulation of the IP_3_ receptors by Ca^2+^ 10,40.
- Mitochondrial NADH concentration
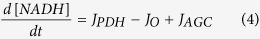
- Mitochondrial ADP concentration
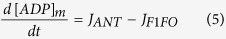
- Cytosolic ADP concentration
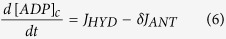
- Voltage difference across the inner mitochondrial membrane (positive corresponding to an excess of positive charges in the cytosol)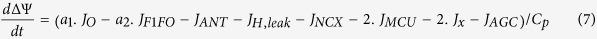 C_p_ is a constant that includes both the membrane capacitance and the Faraday constant.
- Conservation of intracellular Ca^2+^
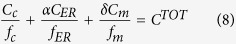
f_ER_ is the Ca^2+^ buffering capacity of the ER.
- Conservation of total (oxidized and reduced) NADH
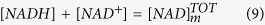
- Conservation of di- and trisphosphorylated adenine nucleotides in mitochondria
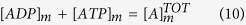
- Conservation of di- and trisphosphorylated adenine nucleotides in the cytosol

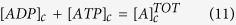


The conservation relations described by Eqs. (10) and (11) do not always hold as adenine nucleotide/Mg^2+^ transporters, known as SCaMCs, mediate net transfer of ADP and/or ATP to the mitochondrial matrix. However, on the short time scales investigated in this study, we expect this transporter to have a minor role since SCaMCs are much slower than the adenine nucleotide translocases (ANT) and have a low affinity for cytoplasmic Ca^2+^ 18.

#### Kinetic expressions for fluxes and reaction rates


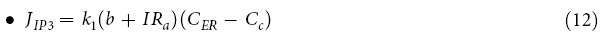


where 
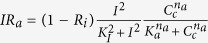


This is a classical expression for the Ca^2+^ flux through the IP_3_ receptor that is rapidly activated at low cytosolic Ca^2+^ and more slowly inhibited at high cytosolic Ca^2+^ 40. It is defined with respect to the volume of the ER. I stands for the concentration of IP_3_ that is taken as a parameter whose value directly reflects the level of cell stimulation.


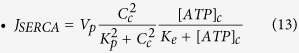


It represents the Ca^2+^ flux through an unidirectional SERCA ATPase, that transports Ca^2+^ from the cytosol into the ER, against the concentration gradient, using the energy provided by the hydrolysis of ATP^41^. It is defined with respect to the volume of the cytosol.

We now describe the kinetic expressions for mitochondrial Ca^2+^ fluxes and metabolism. Most of these processes have been already individually described by complex models quantitatively accounting for energetic-redox mechanisms under various (patho) physiological conditions. Assembling these models would lead to an extremely complex network. Our aim here is to build an intelligible model, centred around Ca^2+^ signalling and its interplay with mitochondrial metabolism. The kinetic expressions used are thus a trade-off between complexity and appropriate conceptualization.


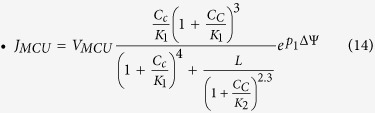


J_MCU_ stands for the flux of Ca^2+^ from the cytosol into mitochondria that occurs through the MCU, defined with respect to the mitochondrial volume. This flux is cooperatively stimulated by cytosolic Ca^2+^ through the MICU1 subunit^42^,^43^. We based this kinetic equation on that proposed by Magnus and Keizer^20^. Although this expression was proposed before the discovery^44^ of the molecular identity of the MCU (previously called the Ca^2+^ uniporter), it is in agreement with the observed properties of this channel. Supplementary Fig. S1 shows the flux through the MCU as a function of cytosolic Ca^2+^ concentration (black curve). This curve, entirely based on the expression and the parameter values proposed by Magnus and Keizer^20^ is in very good agreement with the results of Csordás *et al*. (2013) on the MCU^42^. If the activation by cytosolic Ca^2+^ is removed (K_2_ → ∞), the curve (in red) becomes less sigmoidal and is shifted to the left, as observed for the MICU1 knock-down. As shown by the blue curve, the simplified expression proposed by Bertram *et al*.^28^ does not accurately reproduce the original expression if [Ca^2+^] > 1 *μ*M. Eq. (14) is based on the Monod-Wyman-Changeux formalism for allosteric enzymes^45^ and further assumes that Ca^2+^ cannot bind to the transporter when subunits are in the so-called “tense” form. Concerning the dependence of this rate on the mitochondrial potential, we have simplified the expression used by Magnus and Keizer and simply used Eyring’s theory. Thus, the rate constant of Ca^2+^ transport increases with the potential difference across the mitochondrial membrane in an exponential manner. As explained in the ‘Results’ section, this change was moreover required to account for the observation that energising mitochondria leads to a decreased frequency of Ca^2+^ oscillations^1^,^46^.


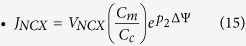


This is the expression proposed by Bertram *et al*.^28^ for the rate of Ca^2+^ extrusion out of mitochondria mediated by the Na^+^/Ca^2+^ exchanger, defined with respect to the mitochondrial volume. This channel exchanges 1 Ca^2+^ for 3 Na^+^ and is thus electrogenic. It is assumed that the cytosolic Na^+^ concentration remains constant and that channel activity is favoured by a large ratio of Ca^2+^ concentrations between the cytosol and mitochondria. As reported previously^47^, we did not consider a possible reverse mode (C_c_ towards C_m_). The dependency on the potential has the same form as for the MCU.


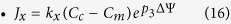


This flux, defined with respect to the mitochondrial volume, was not considered in previous models. Its molecular nature remains to be fully identified (see ‘Results’). We found necessary to consider this flux to account for many experimental results and, in particular, for the fact that C_m_ does not drop in MCU-knocked down cells^42^. The existence of this flux also accounts for the observation that mitochondria take up Ca^2+^ from the cytosol at nanomolar concentrations, at which the MCU is inactive^15^. It is chosen bidirectional to allow for the existence of sustained C_c_ oscillations when the NCX is totally inhibited^33^. If the flux was unidirectional, NCX inhibition would otherwise lead to Ca^2+^ accumulation in mitochondria and arrest of oscillations. As explained in the Results section, we hypothesize that this flux may reflect the low conductance state of the mitochondrial Permeability Transition Pore (mPTP)^48^.


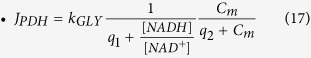


This expression is taken from Bertram *et al*.^28^, who proposed a simplified mathematical expression showing the same behaviour as that derived by Magnus and Keizer (1998)^21^ and taking into account the activation of this enzyme through Ca^2+^-sensitive reversible phosphorylation. This rate not only accounts for the Pyruvate DeHydrogenase(PDH)-catalysed reaction but gathers the glycolytic pathway (k_GLY_) and the Krebs cycle. The Krebs cycle reduces NAD^+^ into NADH, hence the dependency on the adenine nicotinamide ratio. [NAD^+^] is computed from the conservation relationship (Eq. (9)). We have modified the value of 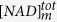 with respect to Bertram *et al*.^28^ to account for experimental observations in HeLa cells^49^. The last factor in Eq. (17) reflects the activation of both the PDH dehydrogenase and the Krebs cycle by Ca^2+^, the latter one occurring at the levels of both the isocitrate- and the *α*-ketoglutarate dehydrogenases. In agreement with experimental data^19^, the K_D_ value for activation by Ca^2+^ (q_2_) is taken equal to 0.1 *μ*M.


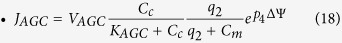


The aspartate-glutamate carrier is part of the MAS NADH shuttle. The two mammalian carriers, aralar and citrin, are activated by moderate cytosolic Ca^2+^ increases (100 nM < S_0.5_ < 350 nM)^18^,^50^. This is taken into account by the second factor in Eq. (18). In many cases, the activation of the MAS pathway is not maintained at Ca^2+^ levels at which the MCU becomes active, although the cross-talk between the Ca^2+^ activation of MAS and the mitochondrial dehydrogenases may vary from tissue to tissue. By activating the Krebs cycle, mitochondrial Ca^2+^ increase would lead to a decrease in the amount of *α*-ketoglutarate, a key-metabolite of the MAS^18^,^50^. To take this limiting step into account in the model, we considered in Eq. (18) that the activity of AGC is inhibited by mitochondrial Ca^2+^ with a threshold value q_2_ that is the same as the threshold for activation of the Krebs cycle by mitochondrial Ca^2+^. As the AGC is electrogenic, J_AGC_ also appears in Eq. (7).


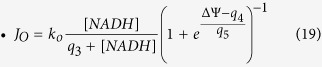


Downstream the Krebs cycle, NADH is then oxidized in the Electron Transport Chain (ETC) to extrude protons from mitochondria. J_O_ thus represents both the rate at which NADH is oxidized (Eq. (4)) and the rate at which H^+^ are extruded. In the rate expression for J_O_ (Eq. (19)), the change of rate with variation in the proton concentration gradient is not considered as in Magnus and Keizer (1997) and Bertram *et al*. (2006)^20^,^28^. Although protons are not considered explicitly in the model, they appear in the evolution equation of the membrane potential (protons extrusion leads to an increase in voltage). In Eq. (7), J_O_ is multiplied by a_1_ to scale the NADH production into a change in voltage due to the proton flux. Eq. (19) is that proposed by Bertram *et al*.^28^ as a simplification of that initially proposed by Magnus and Keizer (1998)^21^. The exponential factor contains the dependency on both the membrane voltage and the proton gradient.


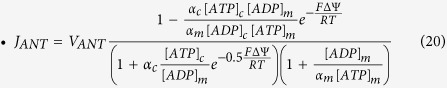


This rather complex expression^20^ describes the activity of a translocator that needs to bind either ATP or ADP on both sides for a conformational change of the carrier to occur. This change allows for a ligand exchange. Thus, 4 possible combinations of ligands are possible (ATP_c_-ATP_m_; ATP_c_-ADP_m_; ADP_c_-ATP_m_; ADP_c_-ADP_m_). The *α*’s stand for the fact that only a fraction of nucleotides has access to the transporter. ADP and ATP are negatively charged (3- and 4-, respectively), which explains the potential dependence of this flux. It is defined with respect to the mitochondrial volume. This equation has been erroneously transcribed and simplified in previous publications^51^. We refer the readers to this reference for a discussion about the usefulness of keeping the original expression.


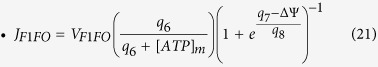


This is the rate of ATP synthesis by the F_1_F_o_-ATPase. This expression, proposed by Bertram *et al*.^28^ accounts for a weak decrease in rate with increasing ATP_m_ (i.e. with decreasing ADP_m_), but for a steep sigmoidal dependency on the mitochondrial potential. ATP synthesis is driven by the proton flux from the cytosol into the mitochondria, which depolarizes the membrane. Thus J_F1FO_ also enters the evolution equation for ΔΨ (Eq. (7)), with the scaling factor a_2_.


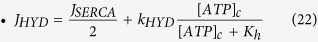


This term represents the rate of ATP consumption in the cytosol. In contrast with previous models, we incorporate the link between Ca^2+^ activity and ATP consumption in the cytoplasm. SERCA pumps (Eq. (13)) are Ca^2+^-ATPases transporting 2 Ca^2+^ ions for one molecule of ATP hydrolysed^52^. The second term of Eq. (22)) encompasses the other ATP-consuming processes in the cytoplasm.


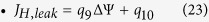


This simplified expression for the ohmic mitochondrial proton leak is taken from Bertram *et al*.^28^

Parameter values are listed in Table 1. These values are the same as those proposed in previous studies when we used kinetic expressions previously published. For new expressions, values were fitted manually to get reasonable agreement with available experimental data. To remain as coherent as possible, we chose data obtained in HeLa cells when available. The full system of equations is simulated using the software package XPPAUT developed by Bard Ermentrout^53^. Bifurcation diagrams are obtained numerically, by solving the differential equations with the MATLAB solver ode23, or ode23tb when using pulses.

### Experiments

#### Cell lines and silencing MCU expression

HeLa cells were from ATCC and were grown in RPMI medium containing 5% fetal calf serum in a 5% CO_2_ incubator. Cells were transfected with a non-targeting siRNA (scrambled) or with two pooled siRNA sequences against the MCU^44^ using the lipofectamine RNAi Max (Invitrogen) following the manufacturer’s instructions. For all experiments MCU mRNA silencing (>70%) was confirmed at 48 h post-siRNA. The rabbit polyclonal antibody against the MCU and Cyclosporin A were from Sigma and siRNAs against the MCU were from Eurogentec.

#### Western-Blot analysis

The equivalent of 4 × 105 cells were scraped in Laemmli-loading buffer. Samples were subjected to Western-blot analysis using the indicated primary antibody followed by anti-rabbit HRP-conjugated antibody. Detection was performed using ECL plus reagent (GE Healthcare Biosciences).

#### Calcium fluorescence microscopy imaging

Analysis of Ca^2+^ variations was performed by loading cells with 3 *μ*M Fluo-4-AM (Invitrogen) in EM buffer containing 120 mM NaCl, 7 mM KCl, 1.8 mM CaCl_2_, 0.8 mM MgCl_2_, 5 mM glucose, 25 mM HEPES pH 7.3, as described previously^54^. Samples were analysed at 33 °C on an inverted Nikon fluorescence microscope, and images were captured every 3 s, using an EM-CCD camera (Hamamatsu), digitized and integrated in real time by an image processor (Compix). All images were corrected for background fluorescence.

## Results

### Ca^2+^ dynamics

**Figure 2:**
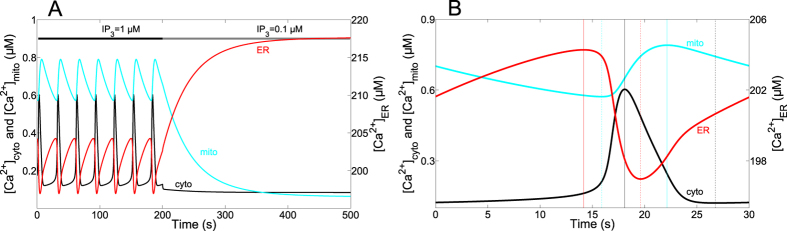
Dynamics of Ca^2+^ exchanges between cytosol, ER and mitochondria. Curves show the simulated changes in Ca^2+^ concentrations in the cytosol (black), the mitochondria (blue) and the ER (red). (**A**) Sustained oscillations triggered by 1 *μ*M IP_3_, followed by the return to a non-stimulated situation (IP_3_ = 0.1 *μ*M). (**B**) Detail of one Ca^2+^ peak occurring when IP_3_ = 1 *μ*M allowing a detailed comparison of the phase relationships in the model and in the experiments of Ishii *et al*. (2006)^31^. Parameter values are listed in Table 1.

Ca^2+^ oscillations occur in the cytosol, the ER and the mitochondria with well-defined phase relationships^31^. We first compared the behaviour of the model defined by Eqs. (1) to (7), with respect to this relationship. Figure 2A shows the evolution of the free Ca^2+^ concentrations in the cytosol (black), the ER (red) and the mitochondria (blue) in the presence of first a relatively high (from 0 to 200 s), followed by a low IP_3_ concentration. The period of oscillations is of the order of 30 s, while their half-width is ~1/5 of the period. A detailed view of one peak is shown in Fig. 2B, where time 0 corresponds to the minimum of Ca^2+^ in the cytosol, during sustained oscillations. From this starting point, Ca^2+^ slowly increases both in the cytosol and in the ER, while C_m_ is still decreasing. Thus, release of mitochondrial Ca^2+^ is responsible for the increase in C_c_, which itself allows the replenishment of the ER. The rise in C_c_ then stimulates the IP_3_R and C_ER_ starts decreasing fast. C_m_ only starts increasing during the fast rising phase of the cytosolic Ca^2+^ peak. Then, C_c_ reaches its maximum, slightly before C_ER_ gets to its minimum value (vertical lines in Fig. 2B). As long as C_c_ is large, the ER refills fast. A change in slope occurs when the rate of C_ER_ increase is imposed by the rate of Ca^2+^ release from the mitochondria into the cytosol. Concerning C_m_, it keeps accumulating quite late after the peak in C_c_. C_m_ finally decreases until the onset of the new cytosolic Ca^2+^ spike, but, interestingly, C_m_ does not recover to basal values during the interspike period. This mechanism is in agreement with the observations of Ishii *et al*.^31^ who observed the same sequence. Thus, mitochondria play an important role in triggering the cytosolic Ca^2+^ spike as they continuously release Ca^2+^ during the silent phase of cytosolic Ca^2+^ oscillations. This effect of mitochondria on Ca^2+^ dynamics is confirmed in the model by the fact that in the absence of mitochondria (J_MCU_ = J_NCX_ = J_x_ = 0), the frequency of cytosolic Ca^2+^ oscillations is lower, but the amplitude is higher. In addition to these modifications, mitochondria also alter the shape of the cytosolic Ca^2+^ peak. Indeed, when J_MCU_ = J_NCX_ = J_x_ = 0, the rate of cytosolic Ca^2+^ decrease is significantly lower (see Supplementary Fig. S2). This is due to the fact that mitochondria slowly release the Ca^2+^ accumulated during the rising part of the oscillation. This more asymmetric shape of the spike is in agreement with experimental observations in HeLa cells^31^.

**Figure 3:**
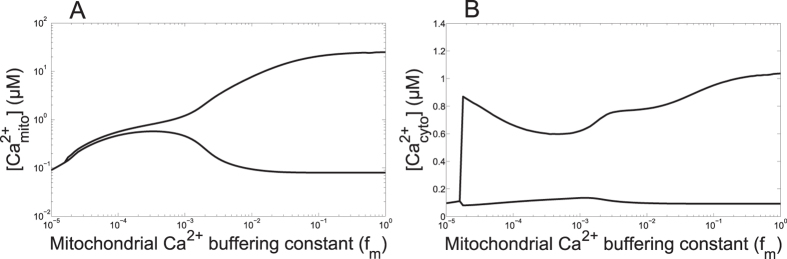
The Ca^2+^ buffering capacity of mitochondria modifies the amplitude of Ca^2+^ oscillations. (**A**) Effect on mitochondrial Ca^2+^. (**B**) Effect on cytosolic Ca^2+^. Curves indicate the minima and maxima reached during oscillations. Parameter values are listed in Table 1. IP_3_ = 1 *μ*M.

In response to a sudden decrease in IP_3_ (Fig. 2A), oscillations rapidly stop in the cytosol, while ER Ca^2+^ evolves towards a high steady value and mitochondrial Ca^2+^ slowly decreases towards its resting state, close to the basal cytosolic Ca^2+^ concentration. This slow decrease in mitochondrial Ca^2+^ down to a basal concentration of about 100 nM is in agreement with observations in HeLa cells^17^,^31^ and chromaffin cells^8^. Reported concentrations of mitochondrial Ca^2+^ during oscillations are highly variable, depending on the study: some experiments report levels in the *μ*M range^8^,^55^ while others describe peaks in C_m_ reaching several tens of *μ*M^2^,^44^. It is highly plausible that results are influenced by the nature of the probe used to monitor Ca^2+^ changes in the mitochondria^56^. However, intercellular and subcellular heterogeneity in mitochondrial Ca^2+^ signalling has been observed using the CEPIA probe in HeLa cells^57^ suggesting that specific characteristics of mitochondria could also explain this difference. In agreement with this hypothesis, we found that the value taken for the Ca^2+^ buffering capacity of mitochondria (f_m_) has a very strong influence on the values of C_m_ associated with changes in C_c_ (Fig. 3A). As shown in Fig. 3B, this factor does not much affect Ca^2+^ changes in the cytoplasm. Thus, different Ca^2+^ buffering capacities in mitochondria due either to intrinsic cell variability or to different concentrations of probes may explain, at least in part, the large range of reported C_m_.

In a next step, we altered the rates of the individual Ca^2+^ fluxes between mitochondria and the cytosol. We first analysed the effect of changing the maximal rate of the NCX. A complete inhibition of the activity of the NCX leads to a decrease in the frequency of the Ca^2+^ spikes (Supplementary Fig. S3). This inhibition indeed leads to a slower Ca^2+^ release from mitochondria and hence to a delayed priming of the IP_3_R to generate the cytosolic Ca^2+^ spike. This decrease in frequency has been observed in HeLa cells stimulated by histamine in the presence of the inhibitor of the mitochondrial Na^+^-Ca^2+^ exchanger CGP37157^33^. The inhibition of the exchanger practically does not change the bifurcation diagram as a function of IP_3_, thus confirming that Ca^2+^ release from mitochondria modulates, but does not cause or inhibit, cytosolic Ca^2+^ oscillations.

**Figure 4:**
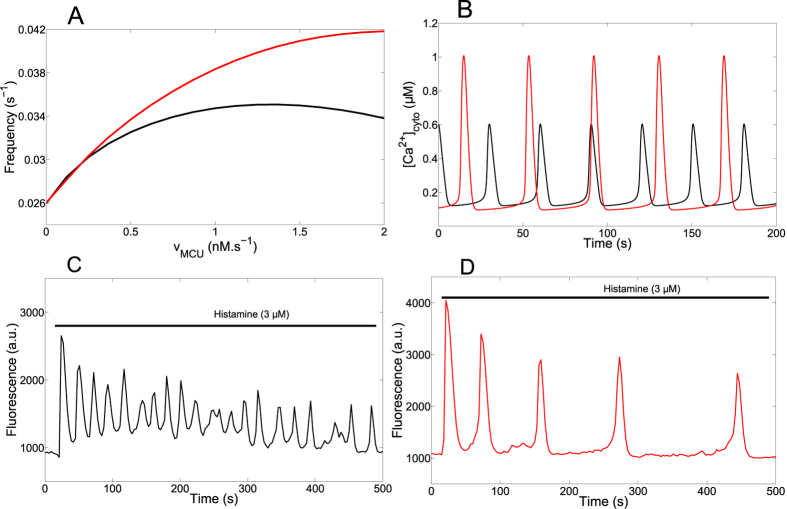
The rate of Ca^2+^ entry into mitochondria alters cytosolic Ca^2+^ oscillations. (**A**) Relationship between the frequency of oscillations and the rate constant of the MCU. The rate constant of the NCX is the default value (black curve, V_NCX_ = 0.35 *μ*M.s^−1^) or is increased (red curve, V_NCX_ = 1 *μ*M.s^−1^). (**B**) Effect of the rate constant of the MCU on cytosolic Ca^2+^ oscillations, as predicted by the model. The black curve shows oscillations for the default value (V_MCU_ = 0.0006 *μ*M.s^−1^) given in Table 1, while the red curve shows oscillations obtained when V_MCU_ = 0. (**C**,**D**) Measurement of Ca^2+^ variations in control (**C**) or MCU-silenced HeLa cells (**D**). Cells loaded with Fluo4 were perfused with 3 *μ*M histamine for the time shown by the black line. Experiments are representative of more than five trials. See also Supplementary Fig. S4.

We next modified the rate constant of the MCU, which is the mitochondrial Ca^2+^ flux opposite to the NCX. As shown in Fig. 4A (black curve), the model predicts a biphasic effect: increasing the activity of the MCU first increases then decreases the frequency of oscillations. At large rates, raising V_MCU_ slightly slows down the oscillations (Fig. 4A, black curve) because mitochondria buffer Ca^2+^ changes in the cytosol. This slows down the ER-cytosol Ca^2+^ exchanges. Surprisingly, at moderate rates the frequency of oscillations decreases when decreasing the rate of Ca^2+^ entry into mitochondria, as also exemplified in Fig. 4B. Indeed, in the range of low values of V_MCU_, Ca^2+^ accumulation in the mitochondria becomes so limited that the subsequent release of Ca^2+^ through the NCX is too weak to boost the IP_3_ receptor. In other words, the amount of Ca^2+^ entering via the MCU becomes the limiting factor in mitochondrial Ca^2+^ handling. In agreement with this explanation, the frequency of Ca^2+^ oscillations is a monotonous increasing function of V_MCU_ when the rate constant of the NCX is increased (red curve in Fig. 4A); in these conditions indeed, mitochondria never slow down the cytosol-ER Ca^2+^ exchanges as the Ca^2+^ sequestered by mitochondria is always rapidly released into the cytosol. This complex relationship between the frequency of Ca^2+^ oscillations and the activity of the uniporter accounts for the observation that activators of the MCU (PPT and kaempferol) stimulate and inhibit the oscillatory behaviour in HeLa cells and in fibroblasts^32^. Interestingly, it is reported in the same studies that the inhibitory effect (i.e. decrease in frequency) is more pronounced if the activity of the NCX is reduced.

That decreasing the rate of Ca^2+^ entry into mitochondria through the MCU slows down Ca^2+^ oscillations appeared as a rather counter-intuitive prediction of the model. We tested this prediction experimentally using HeLa cells transiently transfected with scrambled siRNA or siRNA against MCU (see Supplementary Fig. S4). Indeed, it has been shown that inhibition of the MCU allows to selectively modify the Ca^2+^ uptake capacity of mitochondria without interfering with bioenergetic properties or organelle structure^44^,^58^. The results of a typical experiment are shown in Fig. 4C (control) and D (siRNA anti-MCU). In qualitative agreement with the model, the average period of Ca^2+^ oscillations in response to stimulation by 3 *μM* histamine increases from 31 s (±19 s, n = 63) to 97 s (±39 s, n = 28) in the cells that do not express the MCU. Experiments thus confirmed that MCU inhibition leads to a decrease in the frequency of Ca^2+^ oscillations in HeLa cells. Our interpretation of this observation is that, in the absence of MCU, the very low level of mitochondrial Ca^2+^ does not allow the release of mitochondrial Ca^2+^ between two ER-generated spikes, release that is responsible for the ‘pacemaker-like’ Ca^2+^ trigger of the IP_3_ receptor occurring in control cells.

The increase in period observed in the experiments is, however, much larger than that predicted by the model. We thus investigated if the MCU siRNA treatment shifts the effective dose response to histamine. Supplementary Table S1 shows that this was indeed the case: at 0.3 *μ*M histamine, only 3% of MCU siRNA treated cells displayed a Ca^2+^ response while 39% of control cells oscillated. However, this shift is not the only reason for the observed decrease in period in the cells inhibited for the MCU. Indeed, the period of Ca^2+^ oscillations at the highest histamine dose in anti-MCU siRNA treated cells (10 *μ*M histamine; 98s) is still larger than that seen at the lowest histamine dose in Scrbl siRNA treated cells (0.1 *μ*M histamine; 50 s). Thus, oscillations in the absence of MCU at maximal stimulation, which corresponds to the smallest possible period, are still slower than those obtained at low levels of stimulation in the presence of MCU, pointing to a specific role of the MCU in the control of the frequency of oscillations.

### Possible nature of the bidirectional Ca^2+^ flux between the mitochondria and the cytosol

**Figure 5:**
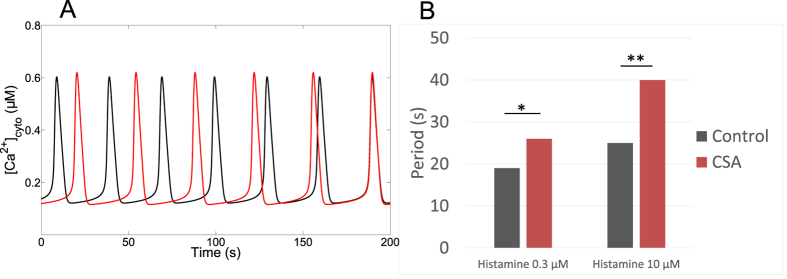
Analysis of the bidirectional Ca^2+^ flux between the cytosol and the mitochondria, J_x_. (**A**) Effect of altering J_x_ on the simulated Ca^2+^ oscillations. The black curve shows oscillations for the default value (k_x_ = 0.008 s^−1^) given in Table 1, while the red curve shows oscillations obtained when k_x_ = 0. In the latter case, the period of oscillations is slightly increased. IP_3_ = 1 *μ*M. (**B**) Experimental investigation of the effect of inhibiting the mPTP with CSA (1 *μ*M) on the period of Ca^2+^ oscillations in Hela cells. n = 64, 18, 16, 14 for control cells (0.3 and 10 *μ*M histamine) and CSA-treated cells (0.3 and 10 *μ*M histamine), respectively. Two groups were compared with an unpaired student’s t-test and two-tail p-value. Results were considered statistically significant when p < 0.05 (*p < 0.05 and **p < 0.01).

In the model, there is another flux allowing a Ca^2+^ exchange between the mitochondria and the cytosol (J_x_, see Eqs. (2) and (16)). This flux was initially incorporated in the model to account for the observation that mitochondria can still take up Ca^2+^ when the MCU is inactive^42^. We found, however, that assuming a reversible flux, the direction of which depending on the electrochemical gradient, led to a better agreement with experimental results as oscillations are maintained when the NCX is inhibited^33^, indicative that Ca^2+^ is extruded from mitochondria by another pathway. For both directions, best agreement is obtained when assuming a low conductance, proportional to the Ca^2+^ concentration gradient. A plausible candidate for this flux is the low conductance mode of the mitochondrial permeability transition pore (PTP)^59^. We analysed the impact of this flux on the simulated Ca^2+^ oscillations. As this flux is bidirectional, it could either boost or slow down Ca^2+^ oscillations. Figure 5A shows that its suppression always decreases the frequency of Ca^2+^ oscillations, as for the NCX. Interestingly, in hepatocytes, an inhibition of the PTP by cyclosporin A (CSA) results in an increase in the interspike interval^60^, in agreement with the behaviour of the model. Experiments also reveal a rise in the mitochondrial membrane potential^60^, which is also observed in the model provided that the flux of protons is reduced, reflecting the fact that the PTP is also permeable to protons.

To further challenge the prediction that the low conductance mode of the mPTP could be involved during cytosolic Ca^2+^ oscillations in HeLa cells, we stimulated CSA-treated cells with histamine. As shown in Fig. 5B, inhibition of the mPTP indeed increased the period of Ca^2+^ oscillations in response to both 0.3 and 1 *μ*M histamine. It should be noted that CSA has been reported to stimulate SERCA pumps in addition to its effect on the mPTP^60^. An increased rate of Ca^2+^ pumping back to ER might thus also participate in the effect shown in Fig. 5B. As in hepatocytes the effect of CSA on the period of Ca^2+^ oscillations was eliminated in the presence of mitochondrial inhibition^60^, we conclude from our observations that the mPTP is a realistic candidate for the bidirectional flux J_x_.

### Mitochondrial variables

**Figure 6:**
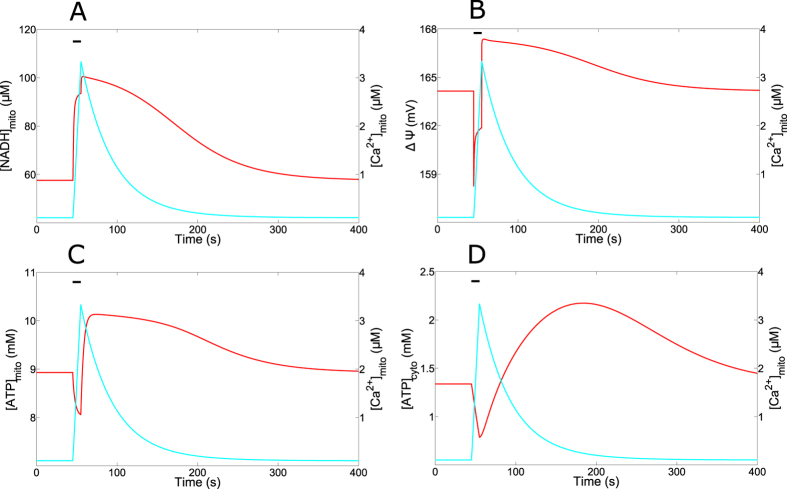
Dynamics of mitochondrial variables. Curves show the simulated changes in the concentrations of the variables related to mitochondrial metabolism in response to a square-wave Ca^2+^ pulse in the cytosol (1.5 *μ*M for 10 s). In all panels, the horizontal line indicates the time of the Ca^2+^ pulse. Mitochondrial Ca^2+^ is shown in blue and the variable indicated on the vertical left axis in red. Parameter values are listed in Table 1.

We next investigated the dynamics of the mitochondrial variables in response to a prototypic Ca^2+^ peak in the cytosol. The Ca^2+^ peak simulated in Fig. 6 is a square wave pulse of 10 s duration and 1.5 *μ*M amplitude. We chose this type of stimulation to optimise the comparison with experimental data. Figure 6A shows the massive and long-lasting (>200 s) increase in NADH resulting from the cytosolic Ca^2+^ spike. It is in agreement with observations in phenylephrine-stimulated hepatocytes^4^. This accumulation of NADH stimulates the Krebs cycle (Eq. (19)) and increases the mitochondrial potential. However, this increase is preceded by a transient decrease in ΔΨ due to the entry of Ca^2+^ from the cytosol into mitochondria (Fig. 6B). Such dynamics for ΔΨ has been observed in HeLa cells stimulated by histamine^34^. The biphasic change in potential induces a similar dynamics of ATP (Fig. 6C) synthesis as the F_1_F_o_-ATPase is highly sensitive to the mitochondrial voltage (Eq. (21)). The initial drop in mitochondrial ATP is also observed experimentally^35^,^61^. In one of these studies^35^, the authors suggested that the drop is a consequence of the initial activation of cytosolic processes, such as those involving ion pumps or contractile proteins, which consume ATP. The drop in mitochondrial ATP would result from its rapid transfer to the cytosol in supply for energy demand. As the increase in NADH occurs later than the rise in Ca^2+^, ATP synthesis is expected to occur later. The model however disagrees with this interpretation. A rise in ATP consumption by the SERCA pumps is indeed observed in the cytosol (Fig. 6D). However, the flux of the translocator (Eq. (20)) is outward for ATP, wich suggests that the initial decrease in ATP can be ascribed to the decrease in ΔΨ.

Figure 6 shows that the rate of decay of [NADH]_m_ is much lower than that of Ca^2+^. Thus, when C_c_ displays sustained oscillations, [NADH]_m_ does not decrease significantly between the spikes, and remains elevated as long as stimulation is maintained (Supplementary Fig. S5). This is due to the slow kinetics of NADH decrease and to the fact that mitochondrial Ca^2+^ does not recover to basal level during the interspike interval (Fig. 2A). The oscillations of NADH on an elevated level are observed in hepatocytes stimulated with high concentrations of phenylephrine^4^.

**Figure 7:**
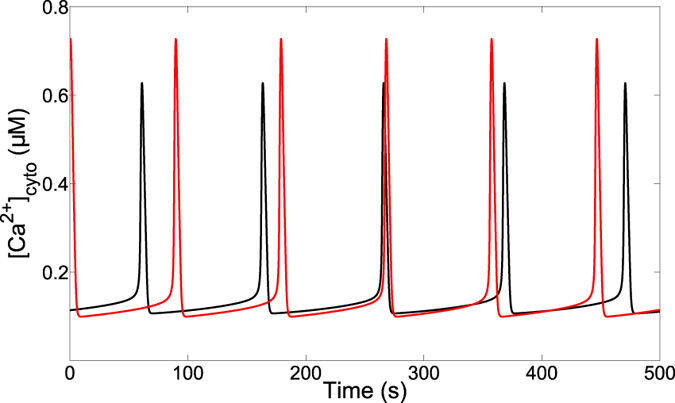
Decreasing the glycolytic input slows down the cytosolic Ca^2+^ oscillations. The curve in black shows cytosolic Ca^2+^ oscillations for the default values of the parameters given in Table 1, while the red curve shows the effect of decreasing the rate of the glycolytic pathway (k_GLY_) to 250 *μ*M.s^−1^. IP_3_ = 0.7 *μ*M.

We next examined how mitochondrial metabolism affects Ca^2+^ oscillations. A classical result in this field is that the frequency of Ca^2+^ oscillations decreases with the amount of mitochondrial substrates^46^. We tested this in the model by decreasing k_GLY_ (Eq. (17)) that represents the input of the glycolytic pathway. As shown in Fig. 7, this leads to a decrease in the period of Ca^2+^ oscillations, as observed experimentally. A less active glycolytic pathway indeed decreases the mitochondrial voltage and thereby reduces the activity of the MCU. In consequence, Ca^2+^ is less actively imported in mitochondria, leading to increased cytosolic Ca^2+^ between successive spikes. This higher levels of interspike cytosolic Ca^2+^ accelerate Ca^2+^ release through the IP_3_R and thus, the next Ca^2+^ spike. To get this result with the model, we needed to impose a rather steep dependence of the MCU on ΔΨ and, in particular, a sensitivity that is larger than that of the NCX (p_1_ > p_2_).

### Robustness of mitochondrial metabolism with respect to Ca^2+^ dynamics

**Figure 8:**
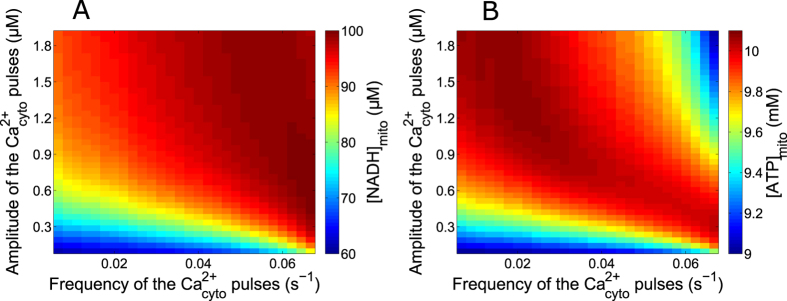
Effect of changing the characteristics of Ca^2+^ spikes on mitochondrial metabolism. The color code indicates the values of [NADH]_m_ (**A**) and [ATP]_m_ (**B**) averaged over one period of the Ca^2+^ repetitive spikes. Baseline Ca^2+^ is set to 100 nM and the duration of the spikes always equals 10 s.

Stimulus-induced Ca^2+^ rises in mitochondria have been suggested as essential for physiological cell bioenergetics^62^,^63^. For such an important function, one expects Ca^2+^ regulation of mitochondrial metabolism to be both versatile and robust. Given the complexity and the highly non-linear character of the regulation of mitochondrial metabolism, this question is best addressed by modelling. Thus, we simulated Ca^2+^ spikes of different frequencies and amplitudes and computed the resulting average values of mitochondrial NADH and ATP concentrations. The results shown in Fig. 8A,B were obtained when simulating artificial square-wave Ca^2+^ pulses lasting 10 s, with a 100 nM basal level of Ca^2+^. We found that maximal average values of NADHm and ATPm were obtained for frequencies and amplitudes of Ca^2+^ spiking that are typically observed in HeLa cells (~0.04 s^−1^ and ~800 nM). Interestingly, optimised metabolism is naturally obtained in the model for the parameter values listed in Table 1, which were independently adjusted to account for published observations on Ca^2+^ and mitochondrial metabolism.

Average [NADH]_m_ is an increasing function of both the frequency and the amplitude of the Ca^2+^ spikes. As shown in Supplementary Fig. S6(A), at low frequencies the minimal values reached during oscillations much depend on the frequency. As NADH decrease is slow (Fig. 6A and Supplementary Fig. S5), [NADH]_m_ does not regain its basal level between the Ca^2+^ spikes, the minimal value reached being fixed by the oscillations’ period. Moreover, AGC also influences this minimal value, as it is activated by low levels of cytosolic Ca^2+^. In consequence, one observes a frequency modulation of the average [NADH]_m_, which is more pronounced with a lower amplitude of the Ca^2+^ spikes. There is a similar amplitude modulation of the average [ATP]_m_, more pronounced with a lower frequency of the Ca^2+^ spikes (Supplementary Fig. S6(C)). In contrast to [NADH]_m_, average values of [ATP]_m_ decrease with large frequency and/or amplitude of the Ca^2+^ spikes. This is due to the decrease in ΔΨ associated with Ca^2+^ entry in mitochondria, which leads to a decrease of the F_1_F_o_ ATPase activity. Consequently, an optimal amplitude and frequency of Ca^2+^ spiking, above which ATP synthesis decreases, can be observed (Fig. 6B and Supplementary Fig. S6 (B and D)).

## Discussion

We are proposing a model for intracellular Ca^2+^ dynamics and mitochondrial metabolism accounting for previously published experimental observations about: (1) the effect of mitochondrial metabolism on Ca^2+^ cycling between ER, cytoplasm and mitochondria during oscillations induced by IP_3_-generating agonists, and (2) the effect of changes in cytosolic Ca^2+^ on mitochondrial metabolism. The model is built from a selection of previously proposed kinetic expressions for the various fluxes and reaction rates, except for the increase of mitochondrial NADH mediated by the malate-aspartate shuttle, which, to the best of our knowledge, has not been considered in previous models. We have modified the expressions for the MCU concerning its dependence with respect to the voltage, and for the SERCA pump, where the ATP requirement was included in the rate equation (Eq. (13)). In a previous study, Fall and Keizer^27^ also modified the original expression of Magnus and Keizer for the MCU^21^. However, these former authors included a dependence of the uptake rate on intra-mitochondrial Ca^2+^, which was not reported in later studies on the MCU^42^,^44^. Concerning the dependence of the SERCA pumps on [ATP], this was introduced in the model for internal coherence. We found that ATP consumption by this pump only slightly affects cytosolic ATP dynamics. Ca^2+^-stimulated production of mitochondrial ATP overtakes this effect by far, in contrast to what has been assumed^35^,^64^.

As compared with previous modelling approaches, we also considered the existence of one additional Ca^2+^ exchange flux between the cytosol and the mitochondria (Eq. (16)). As put forward in some experimental studies, a small influx into mitochondria was found necessary to account for the observed moderate increase in mitochondrial Ca^2+^ when the MCU is inactive^15^,^42^. Similarly, a small flux from mitochondria is required to account for the maintenance of cytosolic Ca^2+^ oscillations when the NCX is inhibited^33^. Given these requirements, a simple assumption is the existence of a passive small flux, whose direction is imposed by the electrochemical gradient. A plausible channel that could mediate this flux is the mitochondrial PTP in its low conductance state. This assumption is corroborated by the observed increase in the period of Ca^2+^ oscillations in CSA-treated HeLa cells (Fig. 5B). Accordingly, a MCU-independent Ca^2+^ influx pathway has been characterized in HeLa cells^65^. In disagreement with our simple expression for J_x_ (Eq. (16)), studies performed in neurons however report that the mPTP can only open in response to a rise in mitochondrial Ca^2+^ 66. On the other hand, the requirement for an efflux pathway other than the NCX has been proposed by Bernardi and Von Stockum^67^, who further assumed that this efflux should correspond to transient openings of the PTP.

Other modes of Ca^2+^ uptake into mitochondria, not considered in the model but reported to be active at low concentrations of cytosolic Ca^2+^, could also play a role. In particular, Ryanodine receptors have been shown to transport Ca^2+^ inside mitochondria in cardiomyocytes and neurons^68^. Also, a rapid mode of Ca^2+^ uptake by mitochondria (RaM) of yet poorly identified molecular nature has been reported in mitochondria isolated from liver and cardiac cells^15^,^69^. The present model could be easily extended to take these fluxes into account (see for example Bazil and Dash (2011))^70^.

The model is fully consistent with the reported effect of alterations in the rates of mitochondrial Ca^2+^ exchanges on the characteristics of Ca^2+^ oscillations. A surprising result of the simulations is that the frequency of oscillations can decrease when inhibiting the activity either of the NCX (Supplementary Fig. S3) or of the MCU (Fig. 4A) despite the fact that these fluxes have opposite directions. This behaviour of the model is confirmed experimentally^33^ (and Fig. 4). It emphasizes the fact that the slow release of Ca^2+^ from mitochondria that occurs between the cytosolic Ca^2+^ spikes paces the oscillations, as suggested by Ishii *et al*.^31^. The increase in the period of oscillations in the absence of MCU is however much larger in the experiments than in the model. This suggests that the absence of MCU alters processes distinct from the oscillatory mechanism and modifies the effective sensitivity to histamine (Supplementary Table S1). In any case, our results confirm that besides IP_3_, mitochondria play a role in determining the interspike interval in addition to other processes more often considered in models, as the time taken by the IP_3_ receptors to recover from Ca^2+^-induced inhibition^71^ or random opening of a sufficient number of channels^11^,^12^.

Altering mitochondrial metabolism in the model also accounts for corresponding published experimental observations about energising mitochondria for example (Fig. 7). Along these lines, we also found (not shown) that decreasing the activity of the ETC (k_o_ in Eq. (19)) increases the frequency of Ca^2+^ oscillations by diminishing the rate of Ca^2+^ pumping into mitochondria, as we have previously reported for Hint2-knock down hepatocytes^72^. Besides, we found that the MAS NADH shuttle participates in the frequency- and amplitude- modulation of Ca^2+^-activated metabolism, by boosting metabolism at low levels of cytosolic Ca^2+^. Thus, the MAS NADH shuttle can increase the amplitude of NADH_m_ oscillations. Interestingly if the effect of the shuttle is not limited when the activity of the MCU becomes significant, NADH oscillations tend to follow cytosolic Ca^2+^ variations and become spiky instead of saw tooth-like as observed experimentally. This *in silico* observation thus confirms the hypothesis that, upon massive activation of the Krebs cycle, *α*-ketoglutarate becomes limiting for the activity of the MAS NADH shuttle^18^.

The model finally predicted that mitochondrial metabolism remains relatively robust with respect to the amplitude and frequency of the stimulating Ca^2+^ oscillations. From a physiological point of view, this property ensures that mitochondrial metabolism filters out the randomness inherent to Ca^2+^ oscillations^11^ to stabilize ATP synthesis, which is a vital process. This was not expected a priori from a modeller’s point of view, given that mitochondrial metabolism is described by highly non-linear kinetic equations. The theoretically predicted robustness of mitochondrial metabolism with respect to changes in Ca^2+^ dynamics is in agreement with MCU knock-down experiments reporting no changes in mitochondrial respiration when MCU expression was reduced^58^. ATP production, however, modestly varies with the dynamical characteristics of the Ca^2+^ spikes, being at large values for periods around 25 s and amplitudes around 800 nM. These values are in the range of those usually seen in HeLa cells, on which our parameter values had been calibrated. Thus, in such a prototypic non-excitable cell, mitochondrial metabolism and Ca^2+^ dynamics are coordinated to optimise bioenergetics.

## Additional Information

**How to cite this article**: Wacquier, B. *et al*. Interplay between intracellular Ca^2+^ oscillations and Ca^2+^-stimulated mitochondrial metabolism. *Sci. Rep.* **6**, 19316; doi: 10.1038/srep19316 (2016).

## Supplementary Material

Supplementary Information

## Figures and Tables

**Table 1:** List of parameter values.

Parameter	Definition	Value	Ref.
a_1_	Scaling factor between NADH consumption and change in membrane voltage	20	This work
a_2_	Scaling factor between ATP production by ATPase and change in membrane voltage	3.43	28
*α*	Volumic ratio between the endoplasmic reticulum and the cytosol	0.1	40
*α*_c_	Factor taking cytosolic ADP and ATP buffering into account	0.111	27
*α*_m_	Factor taking mitochondrial ADP and ATP buffering into account	0.139	27
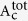	Total concentration in cytosolic adenine nucleotides	2500 *μ*M	73
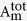	Total concentration in mitochondrial adenine nucleotides	15000 *μ*M	28
b	Ca^2+^ leak from the endoplasmic reticulum	0.01	This work
C_p_	Mitochondrial inner membrane capacitance divided by F	1.8 *μ*M.mV^−1^	28
*δ*	Volumic ratio between the mitochondria and the cytosol	0.0733	28
F	Faraday constant	96480 C.mol^−1^	
f_c_	Fraction of free over buffer-bound Ca^2+^ in the cytosol	0.01	27
f_ER_	Fraction of free over buffer-bound Ca^2+^ in the ER	0.01	27
f_m_	Fraction of free over buffer-bound Ca^2+^ in mitochondria	0.0003	27
k_1_	Rate constant of the Ca^2+^ flux through IP_3_R	30 s^−1^	This work
K_1_	Dissociation constant for Ca^2+^ translocation by the MCU	6 *μ*M	This work
K_2_	Dissociation constant for MCU activation by Ca^2+^	0.38 *μ*M	20
K_a_	Dissociation constant of Ca^2+^ from the activating site of the IP_3_R	0.3 *μ*M	74
K_AGC_	Dissociation constant of Ca^2+^ from AGC	0.14 *μ*M	50
K_e_	Dissociation constant of ATP from SERCA pumps	0.05 *μ*M	41
k_GLY_	Velocity of glycolysis (empirical)	450 *μ*M.s^−1^	28
K_h_	Michaelis-Menten constant for ATP hydrolysis	1000 *μ*M	This work
k_HYD_	Maximal rate of ATP hydrolysis	100 *μ*M.s^−1^	This work
K_i_	Dissociation constant of IP_3_ binding from its receptor	1 *μ*M	54
k_−_	Rate constant of Ca^2+^ dissociation from the inactivating site of the IP_3_ receptor	0.02 s^−1^	74
k_o_	Rate constant of NADH oxidation by ETC	600 *μ*M.s^−1^	28
K_p_	Dissociation constant of Ca^2+^ from SERCA	0.35 *μ*M	54
k_+_	Rate constant of Ca^2+^ binding to the inhibiting site of the IP_3_R	20 *μ*M^−4^.s^−1^	This work
k_x_	Rate constant of bidirectional Ca^2+^ leak from mitochondria	0.008 s^−1^	This work
L	Allosteric equilibrium constant for uniporter conformations	50	21
n_a_	Hill coefficient of Ca^2+^ binding to the activating site of the IP_3_R	3	74
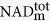	Total concentration of mitochondrial pyridine nucleotides	250 *μ*M	This work
n_i_	Hill coefficient of Ca^2+^ binding to the inhibiting site of the IP_3_R	4	74
p_1_	Voltage dependence coefficient of MCU activity	0.1 mV^−1^	This work
p_2_	Voltage dependence coefficient of NCX activity	0.016 mV^−1^	This work
p_3_	Voltage dependence coefficient of calcium leak	0.05 mV^−1^	This work
p_4_	Voltage dependence coefficient of AGC activity	0.01 mV^−1^	This work
q_1_	Michaelis-Menten-like constant for NAD^+^ consumption by the Krebs cycle	1	28
q_2_	S_0.5_ value for activation the Krebs cycle by Ca^2+^	0.1 *μ*M	This work
	S_0.5_ value for indirect inhibition of the AGC by cytosolic Ca^2+^	0.1 *μ*M	This work
q_3_	Michaelis-Menten constant for NADH consumption by the ETC	100 *μ*M	28
q_4_	Voltage dependence coefficient 1 of ETC activity	177 mV	28
q_5_	Voltage dependence coefficient 2 of ETC activity	5 mV	28
q_6_	Inhibition constant of ATPase activity by ATP	10000 *μ*M	28
q_7_	Voltage dependence coefficient of ATPase activity	190 mV	28
q_8_	Voltage dependence coefficient of ATPase activity	8.5 mV	28
q_9_	Voltage dependence of the proton leak	2 *μ*M.s^−1^.mV^−1^	28
q_10_	Rate constant of the voltage-independent proton leak	−30 *μ*M.s^−1^	28
R	Perfect gas constant	8315 mJ.mol^−1^.K^−1^	
T	Temperature	310.16 K	
V_ANT_	Rate constant of the adenine nucleotide translocator	5000 *μ*M.s^−1^	28
V_AGC_	Rate constant of NADH production via malate-aspartate shuttle	25 *μ*M.s^−1^	This work
V_F1FO_	Rate constant of the F1FO ATPase	35000 *μ*M.s^−1^	28
V_MCU_	Rate constant of the MCU	0.0006 *μ*M.s^−1^	This work
V_NCX_	Rate constant of the NCX	0.35 *μ*M.s^−1^	This work
V_p_	Rate constant of the SERCA pumps	120 *μ*M.s^−1^	54

Fluxes are defined with respect to the volumes of the cytoplasm, the ER or the mitochondria as indicated in the text. Thus, as described in Fall and Keizer^27^, the values of the rate constants obtained experimentally and expressed in nmol.(mg.s)^−1^ are multiplied by the total protein amounts for each compartment and divided by the volume of the compartment.
