# Supplementary material for: Interplay Between Intracellular Ca2+ Oscillations and Ca2+-stimulated Mitochondrial Metabolism
Source: Sci Rep. 2016 Jan 18;6:19316. doi: 10.1038/srep19316 (PMC4725975; doi:10.1038/srep19316)
Supplement: Supplementary Information [file srep19316-s1.pdf]

Interplay Between Intracellular Ca^{2+} Oscillations and Ca^{2+}-stimulated Mitochondrial Metabolism

Benjamin Wacquier[1], Laurent Combettes[2,3], Guy Tran Van Nhieu[4,5,6,7], and Geneviève Dupont[1,*]

[1]Unité de Chronobiologie Théorique. Université Libre de Bruxelles. CP231, Boulevard du Triomphe, 1050, Brussels, Belgium
[2]Université Paris Sud, UMRS1174, Orsay F-91405, France
[3]Institut National de la Santé et de la Recherche Médicale (Inserm), UMRS1174, Orsay F-91405, France
[2]Equipe Communication Intercellulaire et Infections Microbiennes. Centre de Recherche Interdisciplinaire en Biologie(CIRB). Collège de France. 11 Place Marcelin Berthelot, Paris 75005, France
[5]Inserm, U1050, Paris 75005, France
[6]Centre national de la Recherche Scientifique (CNRS), UMR7241, Paris 75005, France
[7]MEMOLIFE Laboratory of excellence and Paris Sciences et Lettres, Paris 75005, France
[*]gdupont@ulb.ac.be

SUPPLEMENTARY INFORMATION

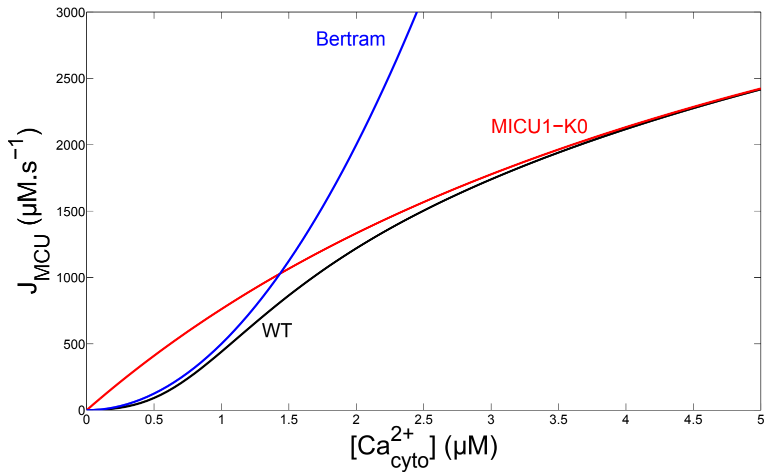

Supplementary Figure S 1. Rate of the MCU as a function of C_c. The black curve shows the flux as given by equ. (14) with the default values of the parameters listed in Table 1. To simulate the effect of the MICU1 mutant (i.e. the MCU that does not express the subunit allowing activation of the channel by cytosolic Ca^{2+}, see Csordás *et al.* 2013), the term C_c/K_2 was set to 0. The blue curve represents the same flux simulated by the expression given in Bertram *et al.* 2006.

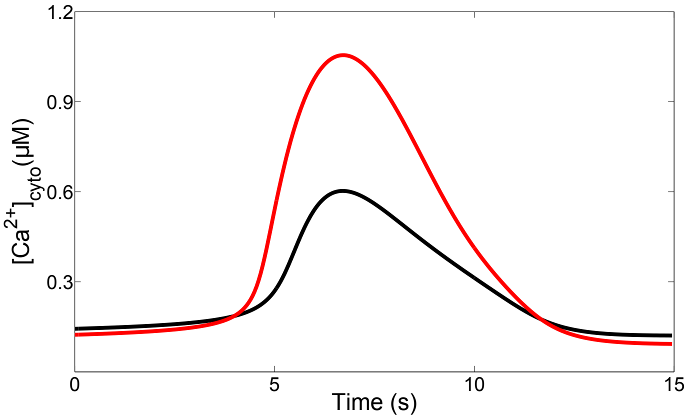

Supplementary Figure S 2. Comparison of the shape of a cytosolic Ca^{2+} peak in the presence (black curve) or in the absence (red curve) of Ca^{2+} exchange with mitochondria. In the presence of mitochondria, peaks are less symmetrical, with a less rapid decrease due to the slow release of Ca^{2+} from mitochondria. Oscillations have been obtained as in Fig.2, except for J_{MCU}=J_{NCX}=J_X=0 for the red curve.

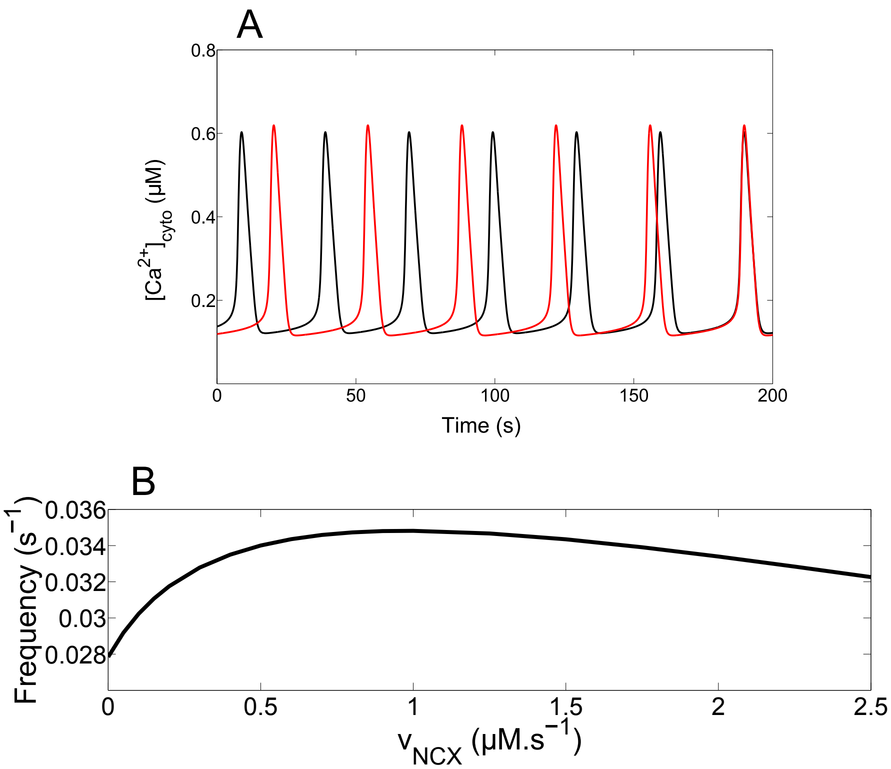

Supplementary Figure S 3. Relationship between the frequency of oscillations and the rate of the Na^+/Ca^{2+} exchanger. The default value in the simulations is 0.35 μM.s^{-1}. In this range, the frequency decreases when decreasing V_{NCX}. At much larger values of this parameter, the frequency decreases with increasing V_{NCX}. Indeed, in such conditions, Ca^{2+} cannot accumulate significantly during cytosolic Ca^{2+} spikes, and thus, cannot be released between the spikes and activate the IP_3 receptor. Parameter values are listed in Table 1. IP_3=1 μM.

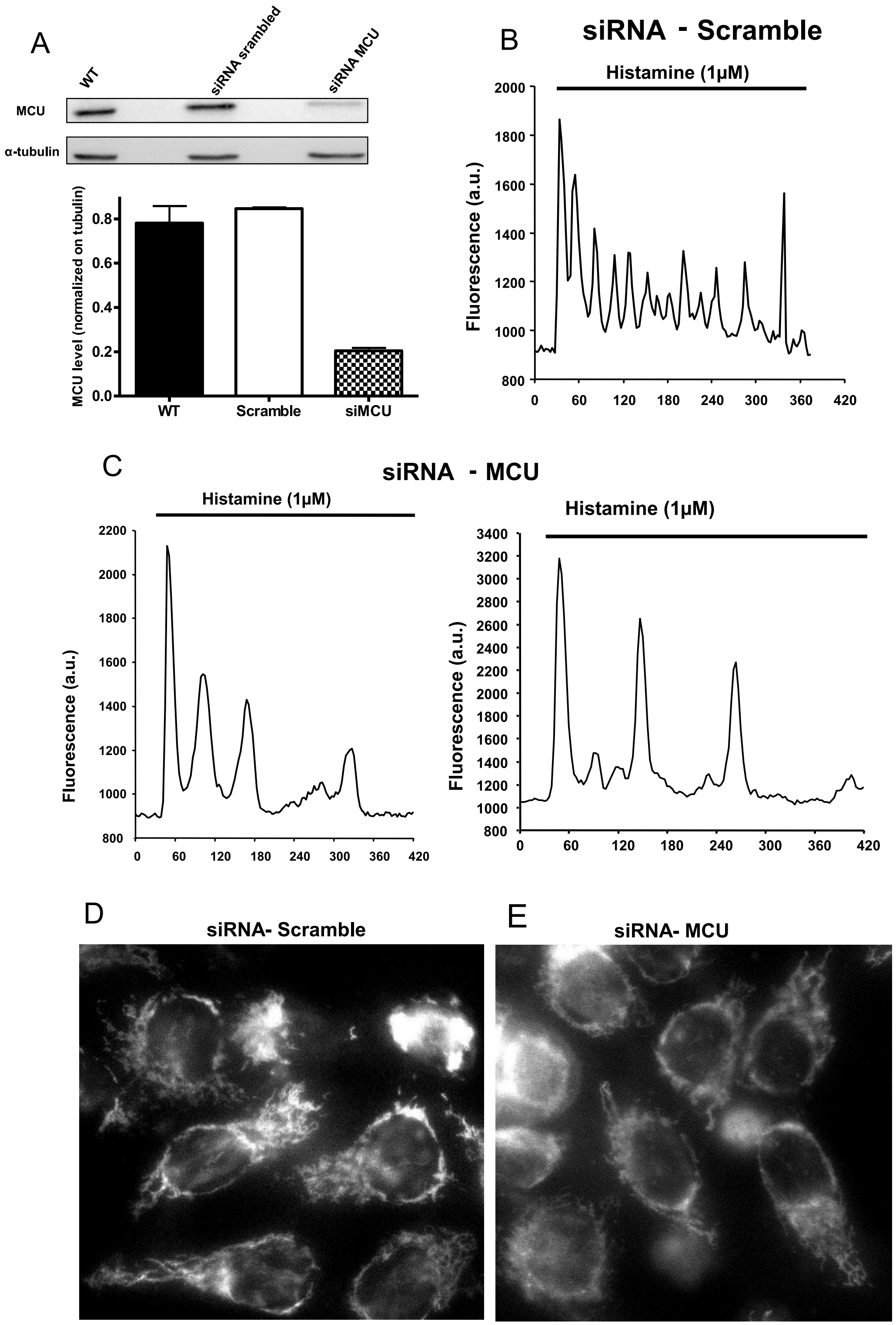

Supplementary Figure S 4. Down-regulating MCU affects cytoplasmic Ca^{2+} oscillations induced by histamine in HeLa cells. Cells were transfected with scramble or MCU-specific siRNA as described in Material and Methods section. Western blot of control, siRNA-scrambled and MCU-specific siRNA transfected cells (revealed with an anti-MCU antibody), is presented in comparison with the housekeeping protein alpha-tubulin (A). The bars represent the average decrease in MCU levels of three different experiments. The endogenous level of MCU was decreased by the siRNA in the whole cell population by approximately 75 %. Histamine (1 μM) induced cytoplasmic Ca^{2+} oscillations in scrambled siRNA (B) or in cells expressing MCU-specific siRNA (C) loaded with Fluo4. Histamine was perfused for the time shown by the black line. At the end of the experiment, incubation of cells with Mitotracker Red CMXRos (1 μM, Molecular Probe) for 5 min, shows that mitochondrial morphology remained roughly unchanged after silencing of MCU (D and E). Experiments are representative of more than five trials.

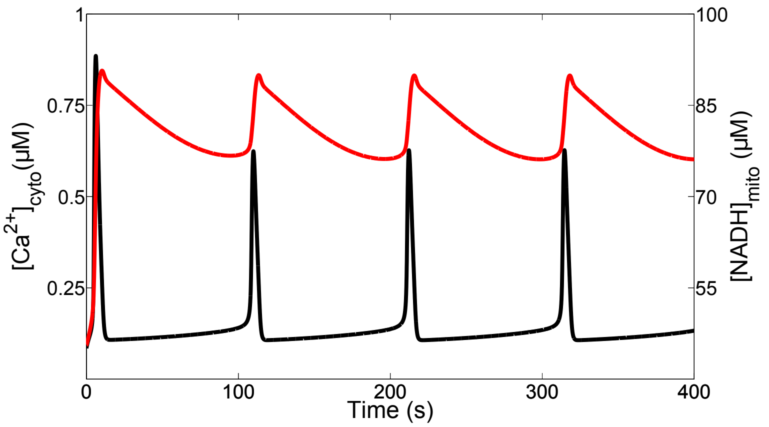

Supplementary Figure S 5. Dynamics of [NADH] during sustained Ca^{2+} oscillations. The variations in the concentration in mitochondrial NADH (red) are shown together with the oscillations of cytosolic Ca^{2+} (black) triggering mitochondrial metabolism. As NADH decrease is relatively slow, NADH remains at high levels as long as oscillations are maintained. Parameters values are listed in Table 1. IP_3=0.7 μM.

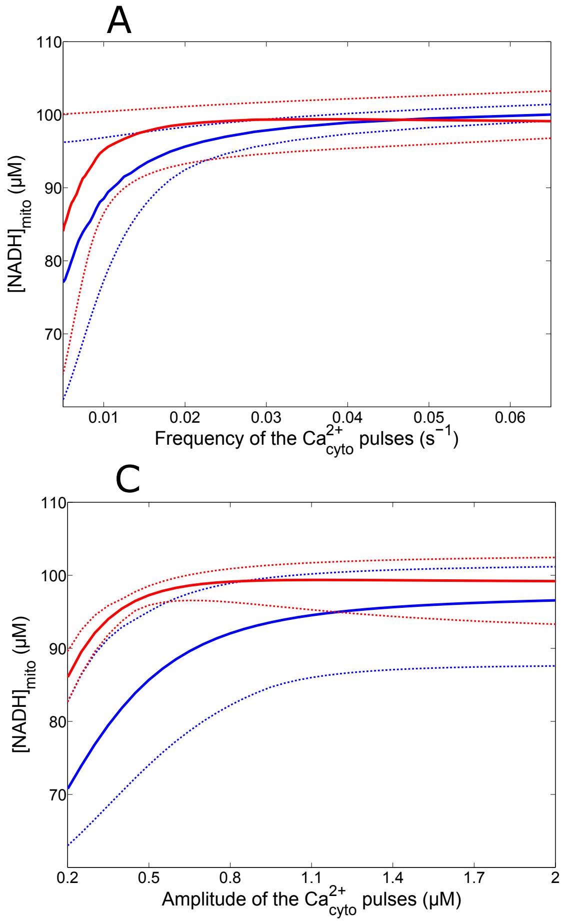

Supplementary Figure S 6. Effect of changing the characteristics of Ca^{2+} spikes on mitochondrial metabolism. Upper panels show the effect of changing the frequency of square-wave Ca^{2+} spikes on the NADH (A) and the mitochondrial ATP (B) levels. Dotted curves indicate maximal and minimal values reached during oscillations, while the plain curve shows the average value. Lower panels show the effect of changing the amplitude of square-wave Ca^{2+} spikes on the NADH (C) and the mitochondrial ATP (D) levels. In panels (A) and (B), the blue curves refer to an amplitude = 0.6 μM and the red curves to an amplitude = 1.2 μM. In panels (C) and (D), the blue curves refer to a frequency = 0.01 s^{-1} and the red curves to a frequency = 0.033 s^{-1}. In all cases, basal level of C_c equals 100 nM and the duration of the spikes equals 10 s.

Supplementary Table S 1. **Histamine sensitivity (% of responding cells) and period of Ca^{2+} oscillations in HeLa cells transfected either with Scrbl siRNA (left) or with siRNA against MCU (right).**

	**Ctrl**		**No MCU**	
	%	Period	%	Period
**0.1 μM**	16.5	49.9 ± 39.5 s	0	
**0.3 μM**	38.8	41.5 ± 20.4	2.6	
**3 μM**	69.9	30.2 ± 18.5	71.8	97 ± 39.3 s
**10 μM**	59.3	25.6 ± 18.1	51.3	98.4 ± 46.6 s
